# Methodological Challenges when Using Routinely Collected Health Data for Research: A scoping review.

**DOI:** 10.23889/ijpds.v9i5.2610

**Published:** 2024-09-18

**Authors:** Lili Wei, Ellen Kuenzig, James Im, Yan Zheng, Taylor McLinden, Scott Emerson, Azza Eissa, Henry Halder, Richard Shaw, An-Wen Chan, William Dixon, Vera Ehrenstein, Astrid Guttmann, Katie Harron, Lars G. Hemkens, Asbjørn Hróbjartsson, Ronan Lyons, Shannon E. MacDonald, Jerry Maniate, David Moher, Irene Petersen, Hude Quan, Sigrún Schmidt, Henrik Sørensen, Shirley Wang, David A. McAllister, Sinéad M. Langan, Eric I. Benchimol

**Affiliations:** 1 The Hospital for Sick Children; 2 Tel Aviv University; 3 Simon Fraser University; 4 BC Centre for Excellence in HIV/AIDS; 5 University of Toronto; 6 Alberta Health Services; 7 University of Glasgow; 8 University of Manchester; 9 Aarhus University; 10 University College London; 11 University Hospital Basel; 12 Odense University Hospital; 13 Swansea University; 14 University of Alberta; 15 University of Ottawa; 16 University of Calgary; 17 Harvard University; 18 The London School of Hygiene & Tropical Medicine

Routinely collected health data (RCD) including electronic health records, disease registries, health administrative data and wearables data are not specifically collected for research purposes. Analysis of these data poses unique methodological challenges that must be addressed when conducting research, particularly as availability and use increase.

This scoping review aimed to identify methodological challenges in research using RCD from existing literature (registered protocol: https://doi.org/10.17605/OSF.IO/EBM4D). We searched 6 electronic databases, including medical, health economics, nursing and psychology research databases, between Jan 2015 and Jan 2023, combining multiple “RCD” and “research” search terms (e.g., epidemiologic, informatics, pharmaceutical research). After screening abstracts and full-texts, we doubly extracted methodological themes, categorizing them into different study stages.

We screened more than 23,000 records and included 430 papers. Bias and confounding were the most common methodological issues identified, discussed in relation to both study design and data analysis. Data quality, including data accuracy, validation, completeness, timeliness and cleaning, also posed substantial challenges, particularly during data processing stage. Record linkage and conducting analyses using distributed health networks also pose unique methodological challenges. Heterogeneity, incorporating social determinants of health and statistical models that address methodological challenges are also described in the literature. External validity and reporting are important considerations for RCD research.

Our review identified several methodological challenges facing researchers using RCD. These issues should be addressed to ensure methodologically sound research. These findings will inform the development of a standardized protocol template and accompanying educational platform aimed at enhancing methodological quality and transparency when conducting research using RCD.

